# *GSTF1* Gene Expression Analysis in Cultivated Wheat Plants under Salinity and ABA Treatments

**Published:** 2014-03

**Authors:** Ali Niazi, Amin Ramezani, Ali Dinari

**Affiliations:** 1 Institute of Biotechnology, Shiraz University, Shiraz 71441-65186, Iran; 2 Department of Medical Biotechnology, School of Advanced Medical Sciences and Technologies, Shiraz University of Medical Sciences, Shiraz, Iran; 3 Department of Life Sciences, Tarbiat Modares University, Tehran, Iran

**Keywords:** ABA, salinity, *GSTF1*, qRT-PCR, wheat

## Abstract

Most plants encounter stress such as drought and salinity that adversely affect growth, development and crop productivity. The expression of the gene glutathione-s-transferases (GST) extends throughout various protective mechanisms in plants and allows them to adapt to unfavorable environmental conditions. *GSTF1 *(the first phi GSTFs class) gene expression patterns in the wheat cultivars Mahuti and Alamut were studied under salt and ABA treatments using a qRT-PCR technique. Results showed that gene expression patterns were significantly different in these two cultivars. Data showed that in Mahuti, there was an increase of transcript accumulation under salt and ABA treatments at 3h, 10h and 72h respectively. In Alamut, however, the pattern of transcript accumulation was different; the maximum was at 3h. In contrast, there were no significant differences observed between the cultivars for *GSTF1* gene expression profiles at three levels of NaCl concentration (50, 100, and 200 mM) or in ABA (Abscisic Acid) treatment. It is likely that difference of gene expression patterns between the cultivars (Mahuti as a salt tolerant cultivar and Alamut as a salt sensitive cultivar) is due to distinct signaling pathways which activate *GSTF1* expression. Lack of a significant difference between the *GSTF1* gene expression profile under salt and ABA treatments suggests that the *GSTF1* gene is not induced by stress stimuli. Of course it is possible that other levels of NaCl and ABA treatments cause a change in the *GSTF1* gene.

## INTRODUCTION

Unfavorable environmental conditions such as drought and salinity stress are among the most common abiotic factors affecting agricultural communities and limiting crop productivity in arable land [1, 2]. Losses of agricultural productivity due to abiotic can reduce average yields by 65% to 87% [3**, **4]. Nowadays, it is well known that abiotic stress can lead to a series of morphological, physiological, biochemical and molecular changes in plant growth and productivity [5]. Conditions of drought and salinity may induce similar cellular damage such as osmotic stress, leading to a disturbance of homeostasis [6] and increasing the production of reactive oxygen species (ROS) [7-9]. However, plants have various extended protective mechanisms that allow them to adapt to unfavorable environmental conditions [6, 10]. Studies show that NaCl stress, can result in the disruption of photosynthetic mechanisms via a combination of superoxide and H_2_O_2_ mediated oxidation [11]. Numerous studies have been carried out to investigate the response of plant cells to saline environments and three basic strategies have been determined as protective mechanisms [12]. These strategies are; the prevention of active Na^+^ influx; wilting of leaves and stems in crops and the expression of genes that regulate tolerance to cellular dehydration [13, 14]. Recent studies show that ABA has pivotal regulatory functions in the plant's responses to drought and salt stresses [14]. As mentioned above, drought and salt stress cause an imbalance between reactive oxygen species (ROS) that are produced by electron transport chains of mitochondria and chloroplasts as sources of ROS in plant cells and antioxidant defense [6, 15, 16]. It has been reported that the existence of ROS is essential for cell signaling pathways [17]. However, high concentrations of these molecules can cause damage to plant cells and tissue [18]. Cellular survival against the toxicity of ROS, requires an array of defense mechanisms, one of them being based on the gene's glutathione-s-transferases (GST). These enzymes catalyze the scavenging of ROS and are renowned as house cleaners that are involved in stress tolerance. Indeed, gluthathioue-s-transferases (GST) catalyze the conjugation of glutathione (GSH) to the electrophilic groups of a wide range of hydrophobic molecules and consequently reduce cellular detoxification [19**-**21]. Results of various studies have shown that the main function of GSTs is providing protection against different stress factors [21-24**]**. Based on nucleic acid and amino acid sequences as well as active site residues, plant GST has been grouped in to several categories including phi, tau, lambda, zeta, theta, dehydro-ascorbate reductase (*DHAR)*, elongation factor 1 gamma (*ef1ᵧ*) and tetrachlorohydro-quinone dehalogenase (*TCHQD*). However, most plant GSTs are plant specific and belong to phi and tau groups [23, 25, 26]. The core aim of this article is an assessment of the changes in *GSTF1* (the first GSTFs class) activity and expression patterns in the wheat cultivars (Mahuti as salt-tolerant Iranian wheat cultivar and Alamut as salt sensitive) under conditions of salt and ABA stress. In addition, we sought to determine the function of *GSTF1* in plant tolerance.

## MATERIALS AND METHODS

**Plant Materials and Growth Conditions:** NaCl is the principal cause of soil salinity stress [12, 27, 28]. This experiment was carried out in a greenhouse to test effects of different salinity levels (0 mM as control, 50, 100, or 200 mM NaCl) and different periods of exposure to salinity (0, 3, 6, 10, 24, 72 h) on *GSTF1* gene expression to determine the *GSTF1* gene in two wheat plants. In this experiment, Mahuti (one of the most salt-tolerant Iranian wheat cultivars), [29] and Alamut (salt sensitive) cultivars were used as samples of cultivated wheat (*Triticum aestivum*, 2n = 6X = 42, AABBDD).

Imbibed seeds were kept in the dark for 24 h at 4^o^C and germinated for 3 days at 22^o^C. Seedlings were then grown hydroponically and irrigated with a modified Hoagland solution [30] When plants had reached the 2–3 leaf stage, salinity treatments (control, 50, 100, or 200 mM NaCl) were used in combination with the Hoagland solution (the control plant sample was not exposed to salinity treatments). All solutions contained CaCl_2_ to maintain a Na^+^:Ca^2+^ ratio below 10:1. In order to investigate the ABA treatment on the *GSTF1* gene, an ABA solution (100 mM) was prepared and sprayed onto the leaf tissue. It should be noted that this experiment was conducted with three individual groups; a salinity treatment, an ABA treatment and a control. Sampling was done after 0, 3, 6, 10, 24, and 72 h from treatments. Tests were done on three biological factors and two technical replicates.

**RNA Extraction and cDNA Synthesis:** Total RNA was extracted using a RNX-Plus buffer (CINNAGEN, Iran). About 100 mg of tissue was ground in liquid nitrogen. The ground powder was transferred to1 ml of RNX-Plus buffer in an RNase-free microtube, mixed thoroughly and then left at room temperature for 5 min. 0.2 ml of chloroform was added to the slurry and mixed gently. The mixture was centrifuged at 13,200 ×*g* at 4^o^C for 15 min, and the supernatant was then transferred to a new tube and precipitated with an equal volume of isopropanol for 15 min on ice. The RNA pellet was washed using 75% ethanol and briefly dried and resuspended in 50 µl of RNase-free water. The purified total RNA was quantified by a Nano-Drop ND 1000 Spectrophotometer (Wilmington, USA). DNase treatment was carried out using Fermentas (Fermentas, Hanover, MD) DNase Kit according to the manufacturer’s instructions. 5 µg of DNase-treated RNA was used for the first strand cDNA synthesis, using 100 pmol oligo-dT (18 mer), 15 pmol dNTPs, 20 U RNase inhibitor, and 200 U M-Mulv reverse transcriptase (all from Fermentas) in a 20µl final volume.

**Quantitative Real-Time PCR Analysis:** The primer was designed using Allele ID 7 software for internal controls and *GSTF1* (AF184059) genes. Wheat elongation factors α (M90077) and β-tubulin (U76745) genes were used as the internal control (whose expression proved not to be influenced by salt and ABA stress) for data normalization [31**, **32**, **33] (Table 1). Relative real-time PCR was performed in a 20µl volume containing 1µl cDNA, 10µl Syber Green buffer (Takara), and 4 pmol of each primer.

 Amplification reactions were carried out in a lineGeneK thermal cycler (Bioer, China) with an initial denaturing of 94^o ^C for 2 min, followed by 40 cycles of 94^o^C for 10 sec, annealing temperature (Ta) of each primer pair for 15 sec and 30 sec of extension at 72^o^C. After 40 cycles, the specificity of each amplification was checked based on melting curves from heating the amplicons from 50^o^C to 95^o^C. All amplification reactions were repeated twice under identical conditions in addition to a negative control and five standard samples.

**Table 1:** Sequences of primers used for real-time PCR amplification and the resulting product size

**Primer**	**Sequence**	**Amplicon** **length** **(bp)**
*GST*-1F *GST*-1R	ATGGAAAACACTAACGTTGTACTC AACTTATAAGCCGAGTTTCTTCTTC	105
*EF-α*-1F *EF-α* -1R	TTTCACTCTTGGAGTGAAGCAGAT GACCTCCTTGACAATTTCTTCATAA	103
*β-Tubulin*-F *β-Tubulin*-R	TGTGGCAACCAGATCGGTGC CATAAGGCCCAGTGCGGACAC	211

To ensure that the PCR products were generated from cDNA rather than genomic DNA, proper control reactions were carried out without reverse transcriptase treatments. For quantitative real-time PCR data, calculations were made for relative expressions of *GSTF1* based on the threshold cycle (CT) method. The CT for each sample was calculated using Line-gene K software and the method cited by Larionov et al. [34]. Accordingly, the fold expression of target mRNAs over the reference values were calculated by the equation 2^-DDCT^ [35], where DCT was determined by subtracting the corresponding internal control CT value from the specific CT of the targets (*GSTF1*), and DDCT was obtained by subtracting the DCT of each sample in the experiment from that of the control sample.

**Statistical Analysis:** Analysis of variance (ANOVA) was used to investigate significant differences in *GSTF1* gene expressions under different NaCl concentrations, ABA treatments and different time courses. Duncan’s multiple range test procedure (SAS 6.12) was used to group NaCl concentrations and ABA treatments or time courses based on average expression levels of the genes of interest in each condition. Pearson's correlation (PC) coefficients were calculated to measure the relationship between gene expressions under the various treatments (including NaCl concentration and ABA treatment) and different times. A paired samples t-test was calculated to determine significant differences in gene expression profiles between the two wheat cultivars. Statistical analysis was done using MINITAB 14 (Minitab, Inc., Pennsylvania, USA) and SAS6.12 software (SAS institute Inc., Cary, NC, USA).

## RESULTS AND DISCUSSION

**Figure 1:**
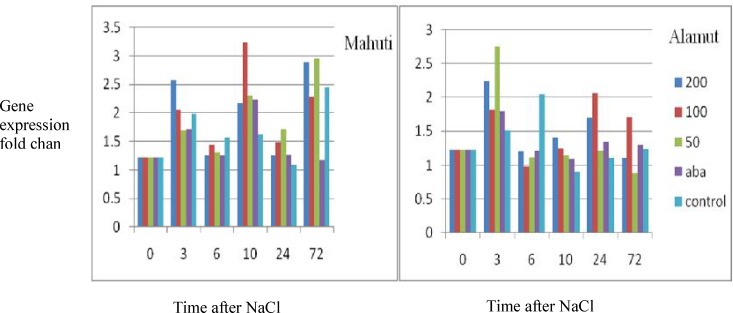
*GSTF1* gene expression pattern under different NaCl concentrations (control, 50, 100 or 200 mM NaCl), ABA treatment and different times after NaCl treatment (0, 3, 6, 10, 24, 72 h).

Quantitative expression patterns for the *GSTF1* gene under treatments (different concentrations of NaCl and ABA treatment) and different time courses are shown in Figure 1. Analysis of variance (ANOVA) showed significant differences between *GSTF1* gene expressions in the two wheat cultivars Mahuti and Alamut under different time courses. Analysis of variance was used to investigate differences in the *GSTF1* gene expression profile under different times, NaCl concentrations and ABA treatments. Significant differences were found between the Mahuti (Mean: 1.769) and Alamut (Mean: 1.407) cultivars (P<0.01).

Mahuti had significantly higher expressions of *GSTF1* at 3, 10 and 72 h compared to other time courses, while Alamut had significantly higher expressions of *GSTF1* at 3 h. In addition, result showed that the *GSTF1* gene expression increased after treatments of ABA and NaCl in both Mahuti and Alamut cultivars (Table 2).

**Table 2:** Duncan's Multiple Range Test for investigation of differences in *GSTF1* gene expression profile under different times after NaCl and ABA treatment in each wheat cultivar

**Time after treatment (h)**	**Alamut**	**Mahuti**
**0**	1.2247^a^	1.2247^a^
**3**	2.0236^b^	2.0039^b^
**6**	1.3119^a^	1.3640^a^
**10**	1.1569^a^	2.3125^b^
**24**	1.4811^a^	1.3596^a^
**72**	1.2452^a^	2.3512^b^

**Note:** In each column means with the same letter are not significantly different

However, the analysis of variance for the investigation of differences in the *GSTF1* gene expression in different wheat cultivars under different NaCl concentrations and ABA treatments showed no significant differences (P>0.05) in the *GSTF1* transcription levels in the two cultivars at 50, 100, 200 mM NaCl and 100 mM ABA treatments.

Evaluation of the relationships between *GSTF1* expression levels in different NaCl concentrations and ABA treatments using the PC coefficient showed no significant correlation between gene expression profiles at 50, 100, and 200 mM NaCl concentrations and 100mM ABA treatment in the cultivars.

It is now clear that GSTs are a diverse group of multi-functional proteins that catalyze a variety of reactions [22, 26**]** Accuracy of the mentioned phrases is supported by results of various studies. For example, diversity of GSTs was investigated and classified in all organisms such as bacteria, animals and plants [22]. As well as, specificity of GSTs isoenzyme functions during stress adaptation and normal condition, are determined. For instance, GSTs can act as GSH transfers, GSH-dependent proxidases and GSH-dependent isomerizes, as well as functioning as non-enzymatic carrier proteins [23, 26]. In general, much research has demonstrated that GSTs gene expression is induced by a broad spectrum of conditions such as biotic and abiotic stress, along with increased GSTs levels and subsequently protects plants against oxidative stress. A wide variety of stress conditions including low temperature, salinity, pathogen attack, and oxidative stress lead to activation of plants’ GSTs gene [16, 24, 36, 37]. In addition, there is evidence that GSTs act as phytohormone-binding proteins, modulating hormone activity and activating the defense signal salicylic acid (SA) [38**-**40].

Designed GSTs phylogenetic tree shows relationships between the major GST classes. The two plant-specific classes, tau and phi have multiple functions in the metabolism of sickness and health in terms of plant condition. The phi class genes mainly involve herbicide detoxification, while the tau class genes include GSTs with hormone responsive ligand in activity. The sequence similarity of phi and tau GSTs is less than 50 percent [22]. Research has shown that the promoter region of tau class GSTs such as Tt *GSTU1* and Tt *GSTU2* has been proven to contain plant hormone responsive elements (ABA, ethylene and auxin) that have a prominent role in stress response [41**,** 42]. In fact the most numerous GSTs class in plants examined refers to tau (GSTUs) genes [43]. Induction of a subset of GSTUs by auxin proved that their activity is prominent for tissue growth. It was also demonstrated that some GSTU genes induced by saline conditions are herbicide softeners [41, 44]. However, other studies on phi GSTs reveal that these genes have subtle changes in metabolite levels [19]. In total this class of GST genes has potential redundancy, that depends on the type of *GSTF*. For example, *GSTF2* was able to bind to auxin compositions, although with a low affinity in each case [45]. Studies on GSTF8 show that plants exposed to biotic and abiotic stresses strongly induced this gene [38, 46**, **47]. In addition, these findings and knowledge about GSTFs function such as *GSTF3*, *GSTF13*, *GSTF14*, are limited [45]. Investigation of *GSTF10* gene expression under drought stress show that this gene was up regulated, but the functional significance of this interaction is unclear [48]. In these experiments *GSTF1* gene expression pattern in wheat leaves under salt and ABA stresses were analyzed to assess its role in plant tolerance. It was observed that gene expression patterns were significantly different in each of the cultivars [6, 49]. Data show that in Mahuti, there was an ascending trend of transcript accumulation at 3h, 6h and 72h and the minimum transcript accumulation at zero time. While in Alamut the pattern of transcript accumulation was different, the maximum transcript accumulation was at 3h. It should be pointed out that significant different between *GSTF1* gene expression profiles were not observed at three level of NaCl concentrations including 50, 100, and 200 mM or in ABA treatments. Finding of researches on transcriptome changes in response to salt, drought and cold stresses demonstrated that there was a kind of genes with common response to various stresses [50-52]. Gene expression studies on the *Suaeda Salsa* *GSTF1* in transgenic rice show that an increase of GST activity was not significant under NaCl and chilling stresses [53]. According to the results, similarity of gene expression pattern of *GSTF1* under salinity and ABA treatment suggested that the targets of common stress-signaling pathways are existence. In the other hand Its seems that differences of gene expression pattern in Mahuti as salt tolerant cultivar and Alamut as a salt sensitive cultivar is due to different signaling pathways which activate *GSTF1* expression. Finally, we can conclude that difference of gene expression pattern in Mahuti and Alamut is a result of different pathways of signaling which activate *GSTF1* expression. Subsequently, after the activation, *GSTF1* product acts in the same pathway to induce downstream genes in two cultivars. In conclusion, it should be noted that little is known about *GSTF1* gene expression and protein function in plant cells, so further studies based on functional genomics and proteomics must be done to clarify these observations.

Ramezani et al. [54] compared the quantitative expression of *TaSOS1* (a transmemberane Na^+^/H^+^ antiporter) and *TaSOS4* genes under same NaCl treatment between Mahuti and Alamut cultivars and were found no significant difference in the level of these two genes transcript accumulation [54]. As Mahuti is one of the most salt-tolerant Iranian wheat cultivars [29], it can be useful to found its tolerance mechanism [29] concluded that salt tolerance mechanism in Mahuti differs from that of other salt-tolerant cultivars like Kharchia). Salt stress is a mixture of ionic, osmotic and oxidative stresses, so the investigation of quantitative expression of other salt tolerance genes (especially the genes of osmotic pathway) in Mahuti may be required. In addition, the amount of Na^+^ in the leaves of Alamut is more than Mahuti [49, 54] so it can affect the gene expression profile of all salt tolerance genes in these two cultivars, including GST. 
